# A Comparison of Psychophysical Dose-Response Behaviour across 16 Sweeteners

**DOI:** 10.3390/nu10111632

**Published:** 2018-11-02

**Authors:** May Wee, Vicki Tan, Ciarán Forde

**Affiliations:** 1 Clinical Nutrition Research Centre (CNRC), Singapore Institute for Clinical Sciences, Agency for Science, Technology and Research (A*STAR), Singapore 117599, Singapore; may_wee@sics.a-star.edu.sg (M.W.); vicki_tan@sics.a-star.edu.sg (V.T.); 2 Department of Physiology, Yong Loo Lin School of Medicine, National University of Singapore, Singapore 117593, Singapore

**Keywords:** sweeteners, sugar reduction, psychophysical dose-response, sweetness growth rate, sweetness potency

## Abstract

Reduction or replacement of sucrose while maintaining sweetness in foods is challenging, but today there are many sweeteners with diverse physical and caloric compositions to choose from. The choice of sweetener can be adapted to match reformulation goals whether these are to reduce calories, lower the glycaemic response, provide bulk or meet criteria as a natural ingredient. The current study sought to describe and compare the sweetness intensity dose-response, sweetness growth rate, sweetness potency, and potential for calorie reduction across 16 different sweeteners including sucrose. Sweetness growth rate was defined as the rate of change in sweetness intensity per unit of sweetener concentration. Sweetness potency was defined as the ratio of the concentration of a sweetener to that of sucrose at equivalent sweetness intensity, whereas the potential for calorie reduction is the caloric value of a sweetener compared to sucrose at matched sweetness intensities. Sweeteners were drawn from a range of nutritive saccharide (sucrose, dextrose, fructose, allulose (d-psicose), palatinose (isomaltulose), and a sucrose–allulose mixture), nutritive polyol (maltitol, erythritol, mannitol, xylitol, sorbitol), non-nutritive synthetic (aspartame, acesulfame-K, sucralose) and non-nutritive natural sweeteners stevia (rebaudioside A), *luo han guo* (mogroside V). Sweetness intensities of the 16 sweeteners were compared with a sensory panel of 40 participants (*n* = 40; 28 females). Participants were asked to rate perceived sweetness intensity for each sweetener series across a range of concentrations using psychophysical ratings taken on a general labelled magnitude scale (gLMS). All sweeteners exhibited sigmoidal dose-response behaviours and matched the ‘moderate’ sweetness intensity of sucrose (10% *w*/*v*). Fructose, xylitol and sucralose had peak sweetness intensities greater than sucrose at the upper concentrations tested, while acesulfame-K and stevia (rebA) were markedly lower. Independent of sweetener concentration, the nutritive sweeteners had similar sweetness growth rates to sucrose and were greater than the non-nutritive sweeteners. Non-nutritive sweeteners on the other hand had higher potencies relative to sucrose, which decreases when matching at higher sweetness intensities. With the exception of dextrose and palatinose, all sweeteners matched the sweetness intensity of sucrose across the measured range (3.8–25% *w*/*v* sucrose) with fewer calories. Overall, the sucrose–allulose mixture, maltitol and xylitol sweeteners were most similar to sucrose in terms of dose-response behaviour, growth rate and potency, and showed the most potential for sugar replacement within the range of sweetness intensities tested.

## 1. Introduction

Sweetness is a key driver of liking in food products and a heightened liking for sweet tastes has been associated with increased intakes of foods with added sucrose [1]. The rising incidence of obesity and type-2 diabetes has been linked with excessive sucrose intake, and fuelled the need for reducing added sucrose in food products [2,3]. Countries such as the United Kingdom and Singapore have pledged to cut sucrose to either 5% free sugars in foods [4] or a 25% sucrose reduction from the current levels [5], namely through reducing added sucrose, using non-nutritive sweeteners and public health education. Sweetness intensity is associated with liking and reducing sucrose can negatively impact the hedonic appeal of a product and consumer acceptance of reformulated products, thereby limiting the widespread reduction of sucrose to achieve these public health goals. Non-nutritive sweeteners can be used to maintain product sweetness, while reducing the negative health impact of excessive sucrose intake, including increased body weight and risk of type-2 diabetes and cardiovascular diseases [6,7,8]. Sweet taste intensity has been shown to be associated with sucrose content of a product, but not with its energy content [9,10] thus creating an opportunity to reduce energy whilst matching sweet taste intensity and liking through the use of lower calorie sweeteners. As such, there has been a rising trend in the use of non-nutritive sweeteners such as sucralose and aspartame, in line with an increasing consumer demand for reduced-calorie foods. In the United States, 1 in 4 consumers include non-nutritive sweeteners in their diets based on a 24-hour diet recall [11]. This may be an effective strategy to improve public health, and a recent meta-analysis has shown that transition to lower-energy sweeteners in place of sucrose leads to reduced energy intake and body weight in both children and adults, as energy reductions associated with the intake of these sweeteners is often not fully compensated for during subsequent eating episodes [7].

Synthetic non-nutritive sweeteners like aspartame and sucralose are still the most widely consumed due to their low cost, quality of their sweet taste and calorie-free advantage, although their long-term metabolic impacts are still being investigated [8]. In addition to reduced sucrose and calories, in recent years there has been a rise in consumer demand for ‘natural’ and clean-label ingredients [11]. As a result, many food manufacturers have shifted towards the use of natural sweeteners such as plant-based glycoside extracts from stevia (rebaudioside, stevioside) and monk fruit (*luo han guo*; mogroside V). Alternative sugars such as the rare sugar d-psicose (allulose) and the isomerized sucrose isomaltulose (palatinose) have also gained interest due to their natural source, clean sweet taste and post-ingestive anti-glycaemic effects [12,13,14]. Polyol sweeteners are a group of sugar alcohols that have been reported to have excellent sweetness quality [15], fewer calories than sucrose and can also act as bulking agents in low sucrose foods, giving them an advantage over several non-nutritive sweeteners [16,17]. To date, the sweetness intensity and dose-response behaviour of many of these more recent sweeteners such as allulose, palatinose, a sucrose–allulose mixture and *luo han guo*, have not been compared alongside sucrose and other sweeteners.

Dose-response relationships have previously been reported for a range of different commercial sweeteners using, for example, the magnitude estimation or spectrum scaling method standardised with reference sucrose solutions [18,19,20,21]. This method obtains relative perceived sweetness intensity values but the comparison to other studies as is highly dependent on the reference solution, scaling method and extent of participant training [21,22]. More recently, psychophysical approaches have compared perceived sweetness intensity using ratings made on the general labelled magnitude scale (gLMS) [23,24,25]. This technique allows for relative comparisons of perceived sweetness intensity between sweeteners across concentrations, and can be useful for determining sweetening capabilities of a novel sweetener in relation to sucrose and other sweeteners [24].

The current study sought to characterise the perceived sweetness intensity of a wide range of different sweeteners to sucrose using a contemporary psychophysical approach. Based on the change in sweetness intensity across a range of concentrations, the dose-response behaviour of each sweetener was compared for their sweetness growth rate, sweetness potency, and potential to support calorie reduction at an equivalent sweetness intensity. These sweetness characteristics can be used as indices to gauge the sweetness and concentration-dependency of a sweetener in relation to sucrose. The sweetness growth rate is the Stevens’ power law exponent in psychophysical terms, and is defined as the slope of the psychophysical relationship describing the rate of change in sweetness intensity with the rate of change in concentration [19]. Sweetness potency was defined as the ratio of the concentration of a sweetener to that of sucrose at an equivalent sweetness intensity [24], whereas the potential for calorie reduction is the caloric value of a sweetener compared to sucrose at matched sweetness intensities. These characteristics were examined across the selected sweeteners to compare sweetener suitability when attempting to reduce or replace sucrose.

## 2. Materials and Methods

### 2.1. Materials

A wide range of different sweeteners was selected to represent a diverse sample of common commercially available sweeteners. The sweeteners used in this study were sucrose (SIS, NTUC Fairprice Supermarket, Singapore), dextrose monohydrate (Suntop Enterprise, Singapore), fructose (Suntop Enterprise, Singapore), allulose (d-psicose; Matsutani Co., Osaka, Japan), palatinose (isomaltulose; Beneo, Singapore), xylitol (Roquette, Lille, France), sorbitol (Suntop Enterprise, Singapore), mannitol (Roquette, Lille, France), erythritol (iHerb, Perris, CA, USA), acesulfame-K (Celanese, Irving, Texas, USA), sucralose (Tate & Lyle, McIntosh, AL, USA), aspartame (Suntop Enterprise, Singapore), *luo han guo* extract (50.6% mogroside V; Hunan Huacheng Biotech Inc., Hunan, China) and stevia (68.0% rebaudioside A; Suntop Enterprise, Singapore). The sucrose–allulose mixture was prepared as a 1:1 blend of sucrose and allulose by weight. Table 1 summarises these sweetener properties including energy density, glycaemic index and bulk properties.

### 2.2. Participants

Forty healthy adult participants (12 males and 28 females; mean age: 26 ± 6 years) were recruited from the campus of the National University of Singapore (NUS) and surrounding areas. Participants were pre-screened for eligibility, basic taste sensitivity and recruitment criteria including being aged between 21 and 50 years old, normal weight (body mass index (BMI) 20–25 kg/m^2^), non-smoker, non-denture wearer, no self-reported sinus, taste or smell dysfunction, not currently following a special diet, no specific food dislikes, allergies or intolerances and not phenylketonuric, diabetic or pregnant. Eligible participants provided informed consent and were compensated for their time. This study (reference: 2017/00787) was approved by the Domain Specific Review Board of the National Healthcare Group, Singapore and complies with the Declaration of Helsinki for research involving human subjects.

### 2.3. Training and Test Procedure

All participants underwent a total of 5 one-hour sessions on separate days, including 1 training session and 4 test sessions. During training, participants familiarised themselves with rating perceived sweetness intensity using the general Labelled Magnitude Scale (gLMS), based on a previously published approach [25]. Participants were asked to rate the overall taste intensity for seven imagined and/or recalled sensations described verbally including the warmth of lukewarm water and the pain from biting their tongue. Thereafter, participants were presented with four basic taste samples and asked to rate sweet (sucrose), salty (NaCl), sour (citric acid) and bitter (caffeine) to ensure they understood how to use the line scale and practice making ratings using the gLMS.

During each test session, participants rated the sweetness intensities of four sweetener sets, with eight samples for each sweetener set. The order of sweetener presented was randomised and balanced across participants and test sessions using a William’s (Latin Square) design. The order of sample presentation within each sweetener set was also randomised. Participants rated a total of 16 sweetener sets over 4 test sessions. For each sweetener set, there is a water sample, six different concentrations of the sweetener (Table 2), and a warm-up sample with a duplicate concentration to one of the samples (sample 4/5; Table 1). The warm-up sample was presented at the beginning to reduce first order effects (data not included in analysis). The concentrations used in this study are by weight basis (% *w*/*v*) presented in Table 2, and the same concentrations expressed in molarity (mmol/L) are provided in the supplementary material (Table S1). For ease of interpretation of the calorie reduction potential and application to product re-formulation, the dose-response behaviour of the sweeteners was expressed on a weight basis in the current study, in line with previous reports [18,21,24]. Therefore, discussions made in this study are based on weight of sweeteners and not by molarity.

All data were collected using Compusense Cloud software as part of the Compusense Academic Consortium (Compusense Inc., Guleph, ON, Canada), in sensory booths under red lights which conform to international standards for the design of test rooms [26]. Participants were instructed to take the sample (15 mL) in their mouth, hold it for 5 s, and rate the sweetness intensity before expectorating. Between samples, participants were instructed to rinse their mouth with filtered water during the mandatory 45-second inter-stimulus interval, to reduce carryover between samples. Solutions were prepared at least 24 h prior to sensory testing using filtered water and stored at refrigeration temperature. The concentration ranges chosen were based on previously published results for each sweetener [23,27,28], and to reflect the sweetness intensities encountered in commercial food and beverage products. Preliminary testing was done to confirm that the sweetness intensities rated for each sweetener were comparable to one another. 

### 2.4. Psychophysical Scaling

Perceived sweetness intensity was rated using a general labelled magnitude scale (gLMS) [22,29,30]. The scale is partitioned by descriptors: no sensation (0), barely detectable (1.5), weak (6), moderate (17), strong (35), very strong (52) and strongest imaginable sensation (100). Individual scaling behaviours for gLMS ratings were standardized within participants using a previously published weight comparison modality matching task [25,31]. All participants were asked to make intensity ratings using the gLMS across a series of different weight stimuli while holding the container on the palm of their non-dominant hand. There was a significant correlation between the overall sweetness ratings and overall mean heaviness ratings (*r* = 0.472, *p* < 0.05). Assuming the intensity ratings of sweetener samples and the heaviness of the bottles were unrelated, the significant correlation indicated that gLMS ratings were due to individual scale-use rather than differences in sweeteners, and thus required standardization across participants. For each participant, a personal standardization factor was obtained using the grand mean for heaviness ratings across weights and participants divided by the individual’s average heaviness ratings. The sweetness intensity rankings for each participant were then multiplied by their individual personal standardization factor to correct for idiosyncratic differences in scale use.

### 2.5. Mathematical Modelling and Data Analysis

#### 2.5.1. Dose-Response Curves

Dose-response curves were fitted using the software Origin Pro 8.1 (OriginLab, Northampton, MA, USA) with the Hill equation for sigmoidal curves:

(1) $$R=R_{\min}+\frac{R_{\max}-R_{\min}}{1+10^{(\log{\mathrm{EC}}_{50}-C)\times \mathrm{HillSlope}}}$$

where R is the predicted sweetness intensity, and C is the sweetener concentration expressed in % *w*/*v*. R_min_ is the minimum sweetness which was constrained to zero, and R_max_ is the predicted maximum sweetness achievable. The midway point between R_min_ and R_max_ is EC50, and the slope of the linear portion of the model is the Hill slope [23]. The fitted parameters are summarised in the supplementary material (Table S2).

#### 2.5.2. Sweetness Growth Rate

Dose-response curves were also converted to log sweetness intensity vs. log concentration plots, which were originally derived from the power law function *R* = kC^n^ in the linear form:

(2) logR=nlogC+logk

where R is the predicted sweetness intensity, C is the sweetener concentration expressed in % *w*/*v*, *n* is the sweetness growth rate (slope of the line or Stevens’ power law exponent), and k is the constant (intercept). The sweetness growth rate provides an overall average index of the rate of change for sweetness intensity with change in sweetener concentration. A sweetener with a steep slope (>1) increases in their perceived intensity at a rate that is faster than changes in concentration, whereas for flatter slopes (<1), greater increases in sweetener concentration are required to produce an equivalent change in sweetness intensity. The log k (intercept) values also refer to the log sweetness intensity of the sweetener at a concentration of 1% *w*/*v*.

#### 2.5.3. Sweetness Potency

Sweetness potency is the ratio of the concentration of sucrose to that of a sweetener at equivalent sweetness intensities (Equation (3)). A sweetness potency of >1 indicates that a smaller concentration of a sweetener is required to achieve the same sweetness intensity at a particular sucrose concentration and, therefore, the sweetener could be considered as ‘more potent’ than sucrose. Sweetness potency is often quoted as a single value e.g., ‘acesulfame-K is 120 times sweeter than sucrose’, however this value should always be reported with the concentration of sucrose at which it was calculated, since sweeteners often have different sweetness growth rates to sucrose.

(3) $$\mathrm{Sweetness}\;\mathrm{Potency}=\frac{\mathrm{Concentration}\;\mathrm{of}\;\mathrm{sucrose}}{\mathrm{Concentration}\;\mathrm{of}\;\mathrm{sweetener}\;\mathrm{at}\;\mathrm{equi}-\mathrm{sweetness}\;\mathrm{intensity}\;\mathrm{to}\;\mathrm{sucrose}}$$

#### 2.5.4. Statistical Analysis

A two-way analysis of variance (ANOVA) was run to confirm absence of first-order and carryover effects. A one-way repeated measures ANOVA analysis was used to test the effect of sweetener type and effect of concentration and statistical significance was set at 5% (α = 0.05). Post hoc pairwise comparisons, using Bonferroni corrections, were used to compare differences in sweetness intensity scores across sweeteners (16 levels) and concentration of sweeteners (6 levels) using the statistical analysis software SPSS (IBM SPSS Statistics for Windows, Version 22.0, IBM Corporation, Armonk, NY, USA).

## 3. Results

**Figure 1:**
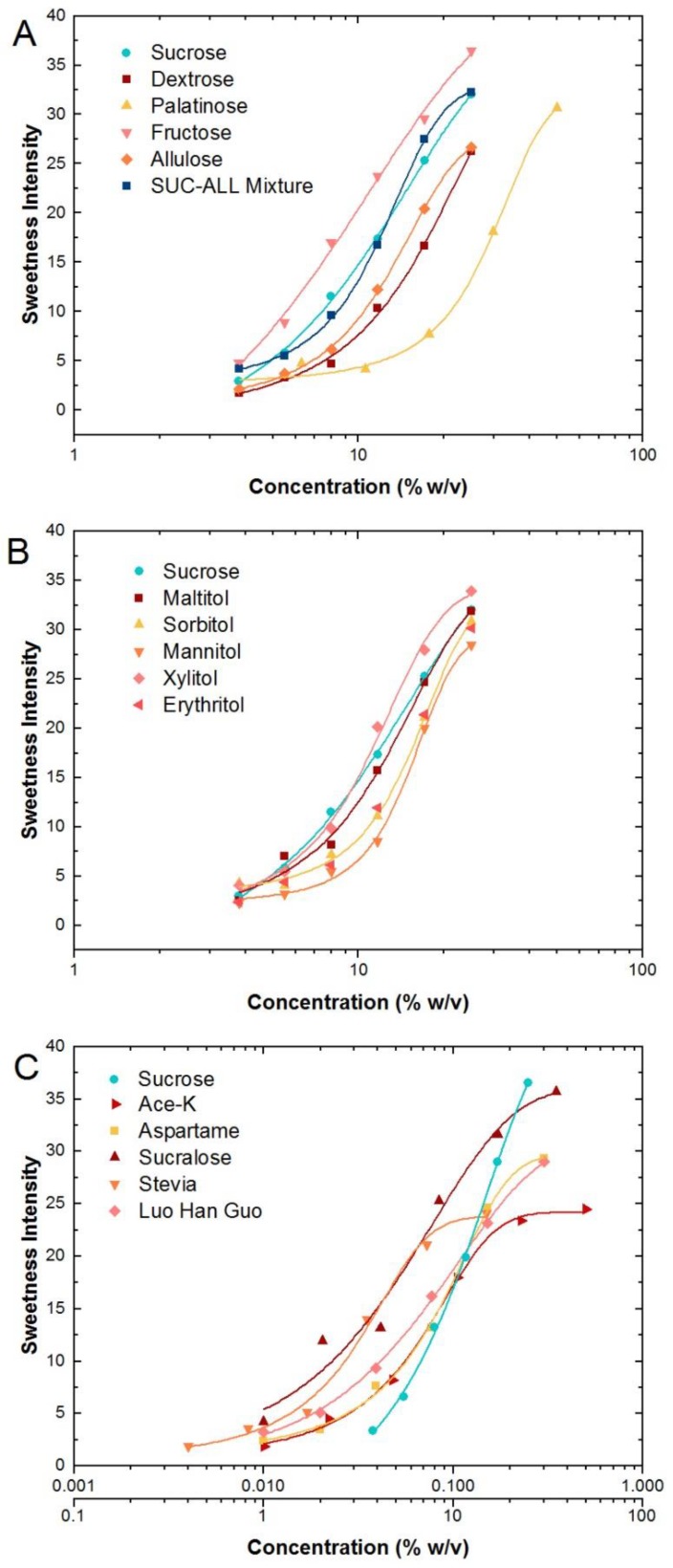
Sweetness intensity with concentration for (**A**) saccharide, (**B**) polyol and (**C**) non-nutritive sweeteners (sucrose is plotted using the secondary x-axis below (0.1–100% *w*/*v*)).

The dose-response for all sweeteners are illustrated on semi-log curves (Figure 1A–C) and fitted with the Hill equation (Equation (1)) with *r*^2^ ≥ 0.95 for all sweeteners. The fitting parameters are listed in Table S2. Repeated-measures ANOVA confirmed that all sweeteners exhibited a concentration dose-dependency for sweetness intensity (*F*_5,39_ = 142.12, *p* < 0.001). Sweetener type had a significant effect on sweetness intensity as concentrations increased (*F*_15,39_ = 18.05, *p* < 0.001) and this was confirmed as a significant interaction between concentration and sweetener type (*F*_75,39_ = 4.20, *p* < 0.001).

### 3.1. Concentration Dose-Response of Sweeteners

The sweetness intensity of sucrose ranged from ‘barely detectable’ (3) to ‘strong’ (33) on the gLMS for the concentration range of 3.8 to 25% *w*/*v*. Nutritive saccharide and polyol sweeteners sucrose, dextrose, allulose, palatinose, maltitol, sorbitol, mannitol, xylitol and erythritol exhibited sigmoidal dose-response functions. By contrast, fructose displayed a more linear response and had a higher sweetness intensity than sucrose and other nutritive sweeteners, across all sucrose concentrations (Figure 1A,B). The sucrose–allulose mixture, maltitol and xylitol had dose-response curves closely matched to sucrose within the range of 3.8 to 25% *w*/*v* sucrose. The dose-response curve for xylitol was similar to sucrose at lower concentrations but had higher sweetness intensity above 11.7% *w*/*v*. Allulose was perceived as less sweet than sucrose at equivalent concentrations, but when allulose and sucrose were blended in a 1:1 mixture, this blend achieved a near identical dose-response curve to sucrose. Palatinose required the highest concentration to match the sweetness intensity of sucrose, and only produced a noticeable rise in sweetness intensity as the concentration went above 10% *w*/*v*. Dextrose, erythritol, sorbitol and mannitol all had lower sweetness intensities than sucrose across the concentration range tested.

Non-nutritive sweeteners exhibited sigmoidal dose-response functions (Figure 1C) and stevia (rebA) and acesulfame-K had flatter responses at low and high concentrations, where increased concentration produced smaller increments in perceived sweetness intensity. In addition, maximum sweetness for these sweeteners peaked below sucrose at ‘moderate’ (25). Sucralose had a higher peak sweetness intensity (35) compared to sucrose (33) at the highest concentration, and was higher than the other non-nutritive sweeteners across equivalent concentrations. Aspartame and *luo han guo* both had similar peak sweetness to sucrose, and their dose-response curves were similar to each other. Their sweetness intensities were weaker than stevia (rebA) at low concentrations (0.01–0.1% *w*/*v*) but stronger at higher concentrations (>0.1% *w*/*v*) when the sweetness intensity of stevia (rebA) plateaued. 

### 3.2. Comparison of Sweetness Growth Rates across Sweeteners

**Figure 2:**
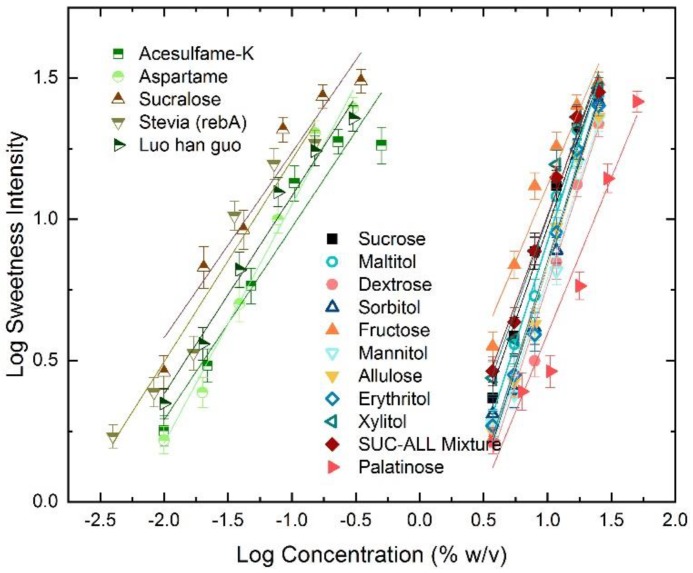
Log sweetness intensity vs. log concentration for 16 sweeteners.

The sweetness growth rate is represented by the slope of the log-log sweetness intensity concentration curves (% *w*/*v* basis) (Figure 2 and Table 3). Sucrose had a sweetness growth rate of 1.31 whereas saccharide sweeteners (dextrose, palatinose, fructose, allulose, sucrose–allulose mixture,) had sweetness growth rates >1, ranging from 1.08 (fructose) to 1.46 (sorbitol). The bulk polyol sweeteners (sorbitol, xylitol, mannitol and erythritol) had sweetness growth rates with similar slopes to sucrose (~1.3–1.4), indicating a similar growth rate to sucrose such that changes in concentration produce similar changes in sweetness intensity. Palatinose and fructose yielded much flatter sweetness growth rates (slopes ≈ 1) amongst the nutritive sweeteners, with 1.10 and 1.08 respectively. By contrast, non-nutritive sweeteners had compressed sweetness growth rates < 1, ranging from 0.65 (sucralose) to 0.84 (aspartame).

### 3.3. Sweetness Potency of Sweeteners Relative to Sucrose

Sweetness potency as well as the concentration of sweetener required to achieve equivalent sweetness intensity to sucrose concentrations at 5%, 10% and 15% *w*/*v* are summarised in Table 4. Saccharide and polyol sweeteners had sweetness potencies <1, with the exception of xylitol (at 15% *w*/*v* sucrose) and fructose. Sweetness potency values for allulose increased from 5% to 15% *w*/*v* sucrose respectively, whereas the sweetness potency for maltitol, xylitol and sucrose–allulose mixture were closer to sucrose across sucrose concentrations, emphasising the similarity of their dose-response functions (Figure 1). Non-nutritive sweeteners had decreasing sweetness potencies at increasing sucrose concentrations. Sucralose has the highest sweetness potency across all sweeteners, but also the largest decline, from sweetness potency of 521 at 5% *w*/*v*, to 201 at 15% *w*/*v* sucrose. Aspartame had the smallest decline in sweetness potency among the non-nutritive sweeteners at higher sucrose concentrations.

### 3.4. Potential for Calorie Reduction across Sweeteners

**Figure 3:**
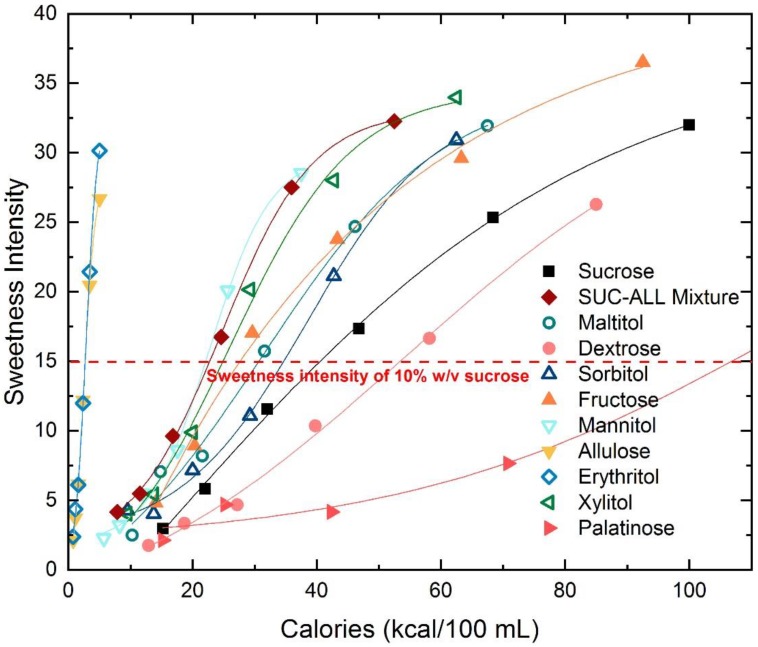
Energy content (kcal/100 mL) of nutritive saccharide and polyol sweeteners to achieve sweetness intensities ranging from weak (6) to strong (35).

Figure 3 shows the caloric value across the different nutritive sweeteners at sweetness intensities ranging from weak (6) to strong (35). The equivalent sweetness intensity to sucrose per unit calorie provides a summary of the calorie saving potential across the different sweeteners. With the exception of dextrose and palatinose, all of the other nutritive sweeteners enable calorie saving at an equivalent perceived sweetness intensity to 10% sucrose (indicated by red line on Figure 3). Allulose and erythritol have the lowest energy densities (0.2 kcal/g) and can achieve an equivalent sweetness intensity to 10% sucrose with very few calories (~95% reduction). For example, a product with 10% *w*/*v* sucrose could potentially be reduced from 40 kcal to <5 kcal/100ml by substituting with allulose or erythritol. Mannitol, sucrose–allulose mixture, xylitol, fructose, maltitol and sorbitol provide about 5–20 kcal/100 mL savings in terms of energy required to achieve equivalent sweetness to 10% sucrose.

## 4. Discussion

In order to support sugar reduction, sweeteners must first match the sweetness intensity of sucrose across the range of perceived intensities commonly encountered in foods and beverages. From a public health perspective, the reduction in sucrose should also support calorie reduction while maintaining consumer appeal beyond sensory-matching perceived sweetness. In addition to their sweetening capacity, sweeteners that can confer additional functionality such as acting as bulking agents, supporting clean labelling or providing an additional anti-glycaemic effect are also highly desirable. A wide variety of sweeteners are currently available and the present study sought to evaluate the sweetening capabilities of these sweeteners in comparison to sucrose based on their dose-response behaviour, sweetness growth rate, sweetness potency and potential calorie savings at equal sweetness intensities.

All sweeteners exhibited sigmoidal dose-response behaviours although fructose displayed a more linear response across the concentration range tested. This sigmoidal relationship between concentration and perceived intensity is the result of the binding kinetics of the sweetener molecules to taste receptors, which plateaus at higher concentration when receptors become saturated [32,33]. From the dose-response curves, all sweeteners were found to match the perceived sweetness intensity of a 10% *w*/*v* sucrose solution, which represents a ‘moderate’ sweetness associated with 10% sugar that is frequently found in many commercially available sweetened and carbonated beverages (e.g., Arizona Ice Tea 10.1 g/100 mL) [34]. This aligns with similar findings from other studies, where the sweetness intensity of ~10% *w*/*v* sucrose was also found to be of ‘moderate’ intensity with rating scores approximately 15 to 20 on the gLMS [23,24,25]. Interestingly, the perceived sweetness intensity of the sucrose–allulose mixture (1:1) was nearly identical to that of sucrose by weight basis, although allulose on its own had consistently lower sweetness intensity than sucrose across the concentrations tested. Previous research has demonstrated that the sweetness intensity of binary mixtures of sweeteners is often an intermediate of the two compounds when tasted alone and at the same total molarity as the mixtures [35,36]. Since the weight of allulose (monosaccharide) is half that of sucrose (disaccharide), the dose-response behaviour of the sucrose–allulose mixture was expected to be between that of sucrose and allulose when expressed in terms of total molarity. Other nutritive sweeteners with smaller molecular weights than sucrose, such as fructose or xylitol, would be relatively even less sweet than sucrose on a molarity basis as compared to weight basis [37]. Nonetheless, for purposes of sweetener application to product re-formulation and interpretation of the calorie reduction potential, the dose-response behaviour of the sweeteners were expressed on a weight basis in the current study, in line with previous reports [18,21,24]. Fructose, xylitol and sucralose were the only sweeteners which had greater peak sweetness intensities than sucrose at the highest concentrations tested, and this has previously been demonstrated across a range of previous studies [23,38,39]. This suggests that ‘high-intensity’ sweeteners such as aspartame and sucralose may be more accurately described as ‘high-potency’ sweeteners, as proposed previously by Antenucci and Hayes [23]. The peak sweetness intensities for the non-nutritive sweeteners acesulfame-K and stevia (rebA) were markedly lower than that of sucrose, reaffirming that these high-intensity sweeteners are not necessarily higher in perceived sweetness intensity than sucrose. Further concentration increments of acesulfame-K, stevia (rebA) and sucralose have been shown to produce a further decrease in sweetness intensity [23,24], which was likely due to bitter taste antagonism at higher concentration among these sweeteners [40]. This decrease in sweetness was not observed for any sweeteners at the concentrations used in the current study. The low peak sweetness of acesulfame-K and stevia (rebA) could limit their use in foods where higher sweetness intensities are required. Nevertheless, it is difficult to determine the true peak sweetness achievable unless a plateau in sweetness can be clearly observed [21], on the condition that the intensity scaling method is not limited by a ceiling effect [29,41]. The concentration ranges for the sweeteners were selected prior to the study based on literature and preliminary experiments, although we acknowledge that further concentration increments would likely result in greater perceived sweetness for some sweeteners. In this case, comparing the sweetness growth rate would be a better indicator of the dose-response trajectory rather than the peak sweetness of each sweetener, to understand whether they are likely to match or surpass the sweetness of sucrose.

The sweetness growth rate is the Stevens’ power law exponent or slope of the log relationship between changing concentration and the perceived sweetness. It should be noted that the sweetness growth rate obtained in this study is a product of the concentration ranges from which they are derived. These range effects mean that sweetness growth rates can change to be higher or lower depending on the range of concentrations tested, and a higher sweetness growth rate is obtained with a smaller concentration range [42]. Sucrose had a sweetness growth rate of 1.3 which is consistent with previous findings which reported sweetness growth rates of 1.15 to 1.3 [18,19,20,24]. The sweetness growth rates of sucralose, stevia (rebA), dextrose and mannitol were also found to be comparable to those previously reported [18,24] and collectively these findings highlight that sucrose, and other nutritive sweeteners exhibit sweetness growth rates greater than non-nutritive sweeteners. This appears counterintuitive since only a small quantity of non-nutritive sweetener is required to impart an intense sweetness, which should be perceived as a higher growth rate. However, sweetness growth rates are based on sweetness intensity changes *per* unit log-concentration, and in relative terms greater quantities of non-nutritive sweeteners are required to achieve a proportional increment in perceived sweetness. This may also be due to the emergence of bitter side-tastes and taste–taste antagonism at higher concentrations [40], or different binding mechanisms across sweeteners, which often remain poorly understood [43]. Sweeteners with lower sweetness growth rates to sucrose are capable of matching the sweetness intensity, albeit over a limited concentration range. The implication is that sweetness growth rates should be considered when estimating the predicted sweetness intensity of a sweetener at concentrations beyond those reported in the dose-response curves. For example, sucralose matched the sweetness intensity of sucrose to an upper concentration of 25% *w*/*v*, but displayed a smaller growth rate, suggesting that the peak sweetness intensity for sucralose would be lower than that of sucrose. This is further supported by the flatter dose-response curve for sucralose at higher concentrations (0.172–0.350% *w*/*v*).

Sweeteners with the same growth rate as sucrose will increase in perceived sweetness intensity with equal increases per unit concentration. Sweetness potency or relative concentration of sweetener required to produce an equi-intense sweetness to sucrose would, therefore, be similar across a range of different concentrations. By contrast, sweeteners with growth rates that differ significantly from sucrose would have sweetness potencies that vary with sucrose concentration, as demonstrated previously [19,20,24,44]. While sweetness growth rate and sweetness potency are closely related indices, sweetness potency is more often used as a quick indication of the quantity required to achieve an equi-intense sweetness to sucrose at a given concentration. Non-nutritive sweeteners have growth rates significantly lower than sucrose, and therefore their sweetness potency is also highly concentration-dependent. The sweetness potency values reported for non-nutritive sweeteners in the current trial were not fully consistent those reported previously. For example, aspartame was found to be 128 and 185 times more potent than sucrose at 5 and 10% sucrose equivalence by Tunaley, Thomson and McEwan [45], and Cardello et. al. [20] respectively, as compared to the 173 (5% *w*/*v*) and 121 (10% *w*/*v*) found in our study. Differences were also found for sweetness potencies of stevia (rebA) and sucralose [24]. Sucrose–allulose mixture, xylitol and maltitol have sweetness potencies closest to 1, indicating that the quantities required to achieve an equivalent sweetness intensity on a weight basis are similar to sucrose. This is an important consideration when the replacement sweetener is also required to substitute some of the bulking properties of the removed sucrose. When calorie reduction without the addition of bulk is the main goal of sucrose reduction, low calorie and/or high potency sweeteners may be more effective, particularly among certain products (i.e., beverages) as lower concentrations are required to match sweetness intensity.

With the inclusion of several low-calorie nutritive sweeteners in the study, it is still possible to achieve calorie reduction while maintaining sweetness, even when sweetness potency was not equivalent or higher than sucrose. With the exception of dextrose and palatinose, the nutritive sweeteners profiled supported reductions in total calories to varying extents while meeting the equivalent sweetness intensity of a 10% sucrose solution. Allulose and erythritol in particular have the lowest calories at a sweetness intensity equivalent to 10% sucrose, and could be used to support substantial calorie savings. When allulose was mixed 1:1 to partially replace sucrose, the sucrose–allulose mixture showed very similar sweetening properties to sucrose, while supporting significant reduction in overall calories. Considering the sweetness intensity, sweetness growth rate, sweetness potency and potential calorie reduction together, the sucrose–allulose mixture, maltitol and xylitol were most similar to sucrose, across the concentrations studied. All three sweeteners can provide bulk, support a clean label, reduce total calories for equivalent sweetness intensity and in the case of sucrose–allulose mixture, also impart an additional anti-glycaemic effect post-ingestively [12,14]. When selecting the appropriate sweetener for use in sugar reduction, the sensory, physical, nutrient and metabolic impact of the selected sweetener should be considered, and in some cases sweeteners will have desirable characteristics for some properties but not others. For example, palatinose has an anti-glycaemic benefit which is desirable for products that support the management of glucose homeostasis, but it is required at a greater concentration and energy content to achieve an equi-intense sweetness intensity to sucrose [13]. Non-nutritive sweeteners are calorie-free but may have certain undesirable side-taste attributes, especially at higher concentrations [23,24,40,46] which may limit their usage. With these factors considered, it may be judicious to blend sweeteners with sucrose to optimise the sensory profile and sweetening capacity, and compromise on some elements of the nutrient or metabolic profile. Results from the current study demonstrate that blends like the sucrose–allulose mixture provide encouraging results with excellent sweetness characteristics in line with sucrose, at a fraction of the calories and a potential post-consumption anti-glycaemic benefit.

Findings from the current study are aligned with previously reported differences in sweetness dose–response, growth rate and potency, although some subtle differences were observed in the absolute values reported. These are likely to be due to differences in methodological approach, individual variability, sweetener source, matrix effects, concentration range used, pH and temperature [20,21,23,25,47]. Our findings are most closely aligned with those previously reported by Antenucci and Hayes which were collected using the same standardised gLMS approach to rate sweetness intensity. This approach minimises ceiling effects and produced comparable intensity ratings for many of the same sweeteners [23]. There is currently no official standardised approach to quantify the perceived sweetness intensity of a sweetener, although the comparability of results would be greatly enhanced if future efforts adopt a consistent objective approach, such as used in the current and previous studies [23,24,25].

In choosing to focus on sweetness intensity alone, we have not accounted for other temporal and qualitative taste differences between the sweeteners reported elsewhere [46]. In addition, perceived sweetness intensity rated in water does not account for matrix effects or taste–taste interaction that would occur when these same sweeteners and concentrations are used in foods and beverages [48,49]. The current findings present an overview of the psychophysical dose-response behaviour of a wide range of different sweeteners, and provides guidance on the similarity of various sweeteners to sucrose and the likely calorie savings that could be achieved if they are used to reduce or replace sugar. Future research should aim to extend this further by profiling the temporal and qualitative differences between sweeteners and characterising their performance in food and beverages. 

## 5. Conclusions

The current paper characterized the psychophysical dose-response behaviour of 16 sweeteners and identified differences in the peak sweetness, sweetness potency and sweetness growth rate. Sucrose—allulose mixture, maltitol and xylitol exhibited similar psychophysical behaviours to sucrose in terms of peak sweetness intensity, sweetness growth rate and sweetness potency, and showed the greatest potential to match the sweetness of sugar, for significantly fewer calories. Non-nutritive sweeteners offer significant calorie savings, but had lower peak sweetness intensities and lower sweetness growth rates, which may not limit their ability to match sweetness intensity over a wider range of sucrose concentrations. Differences in the psychophysical relationships identified in the current paper should be considered when selecting sweeteners to support sucrose reduction or replacement, and offer significant opportunities to match the perceived sweetness of sugar, while supporting energy density reductions.

## Figures and Tables

**Table 1:** Characteristics of the 16 sweeteners used.

Sweetener	Kcal (kcal/g)	Glycaemic Index	Provides Bulk	High Potency	Natural
Acesulfame-K	0.0	0		✓	
Allulose	0.2	0	✓		✓
Aspartame	4.0	0		✓	
Dextrose	3.4	100	✓		✓
Erythritol	0.2	0	✓		✓
Fructose	3.7	19–23	✓		✓
*Luo han guo*	0.0	0		✓	✓
Maltitol	2.7	36	✓		
Mannitol	1.5	0	✓		
Mixture ‡	2.1	-	✓		✓
Palatinose	4.0	32	✓		✓
Sorbitol	2.5	9	✓		
Stevia (RebA)	0.0	0		✓	✓
Sucralose	0.0	0		✓	
Sucrose	4.0	60	✓		✓
Xylitol	2.5	13	✓		

‡ 1:1 sucrose−allulose mixture (weight basis). A ✓ indicates that the sweetener belongs to the respective category.

**Table 2:** Concentrations tested for each of the sweetener set by weight basis (% *w*/*v*).

Sweetener	Abbreviation	Concentration (% *w*/*v*)						
		Sample 1	Sample 2	Sample 3	Sample 4, 5	Sample 6	Sample 7	Sample 8
Acesulfame-K	(ACE)	0	0.0100	0.0219	0.0478	0.105	0.229	0.500
Allulose	(ALL)	0	3.80	5.50	8.00	11.7	17.1	25.0
Aspartame	(ASP)	0	0.0100	0.0197	0.0390	0.0770	0.152	0.300
Dextrose	(DEX)	0	3.80	5.50	8.00	11.7	17.1	25.0
Erythritol	(ERY)	0	3.80	5.50	8.00	11.7	17.1	25.0
Fructose	(FRU)	0	3.80	5.50	8.00	11.7	17.1	25.0
*Luo han guo*	(LHG)	0	0.0100	0.0197	0.0390	0.0770	0.152	0.300
Maltitol	(MAL)	0	3.80	5.50	8.00	11.7	17.1	25.0
Mannitol	(MAN)	0	3.80	5.50	8.00	11.7	17.1	25.0
Mixture ‡	(MIX)	0	3.80	5.50	8.00	11.7	17.1	25.0
Palatinose	(PAL)	0	3.80	6.30	10.6	17.7	29.8	50.0
Sorbitol	(SOR)	0	3.80	5.50	8.00	11.7	17.1	25.0
Stevia	(STE)	0	0.00400	0.00830	0.017	0.0352	0.0727	0.150
Sucralose	(SCL)	0	0.0100	0.0204	0.0415	0.0844	0.172	0.350
Sucrose	(SUC)	0	3.80	5.50	8.00	11.7	17.1	25.0
Xylitol	(XYL)	0	3.80	5.50	8.00	11.7	17.1	25.0

‡ 1:1 sucrose–allulose mixture (weight basis).

**Table 3:** Slope and y-intercept values of linear fit between log sweetness intensity and log concentration (% *w*/*v*).

Sweetener.	Slope (*n*)	Y-Intercept (log k)
Acesulfame-K	0.68	1.65
Allulose	1.41	−0.58
Aspartame	0.84	1.90
Dextrose	1.40	−0.63
Erythritol	1.45	−0.6
Fructose	1.08	0.04
*Luo han guo*	0.70	1.78
Maltitol	1.42	−0.51
Mannitol	1.38	−0.59
Mixture ‡	1.24	−0.23
Palatinose	1.10	−0.51
Sorbitol	1.46	−0.63
Stevia	0.71	1.93
Sucralose	0.65	1.89
Sucrose	1.31	−0.33
Xylitol	1.30	−0.29

‡ 1:1 sucrose–allulose mixture (weight basis).

**Table 4:** Concentrations matching for equi-sweetness and sweetness potency of 15 sweeteners to 5%, 10% and 15% *w*/*v* sucrose.

Sweetener	Equi-Sweet Concentrations (% *w*/*v*)			Sweetness Potency		
	5% SUC	10% SUC	15% SUC	5% SUC	10% SUC	15% SUC
Acesulfame-K	0.0293	0.0832	0.170	171	120	88.1
Allulose	7.1	13.3	18.9	0.71	0.75	0.80
Aspartame	0.0290	0.0827	0.134	173	121	112
Dextrose	7.8	15.5	21.6	0.64	0.64	0.69
Erythritol	6.9	13.3	17.8	0.72	0.75	0.84
Fructose	4.0	7.4	11.2	1.25	1.36	1.34
*Luo han guo*	0.0191	0.0694	0.141	262	144	106
Maltitol	5.6	11.2	15.8	0.93	0.89	0.95
Mannitol	8.6	14.6	18.6	0.58	0.68	0.81
Mixture ‡	5.0	10.7	14.3	0.99	0.93	1.05
Palatinose	12.7	26.4	34.6	0.39	0.38	0.43
Sorbitol	6.3	13.7	17.9	0.80	0.72	0.83
Stevia	0.0144	0.0395	0.0828	348	253	181
Sucralose	0.0096	0.0387	0.0748	521	258	201
Sucrose	5	10	15	1	1	1
Xylitol	5.1	9.9	13.3	0.98	1.01	1.12

‡ 1:1 sucrose–allulose mixture (weight basis).

## Supplementary Materials

The following are available online at http://www.mdpi.com/2072-6643/10/11/1632/s1, Table S1: Concentrations tested for each of the sweetener tastant series in molarity (mmol/L), Table S2: Fitting parameters for dose-response of sweeteners using the Hill equation.
