# Supplementary material for: A Comparison of Psychophysical Dose-Response Behaviour across 16 Sweeteners
Source: Nutrients. 2018 Nov 2;10(11):1632. doi: 10.3390/nu10111632 (PMC6266678; doi:10.3390/nu10111632)
Supplement: Supplementary file 1 [file nutrients-10-01632-s001.pdf]

Supplementary Materials

Table S1. Concentrations tested for each of the sweetener sets in molarity (mmol/L)

Sweetener	Molecular Weight (g/mol)	Concentration (% *w/v*)						
		1	2	3, 4	5	6	7	8
Acesulfame-K	201.2	0	0.50	1.09	2.38	5.22	11.38	24.85
Allulose	180.2	0	210.9	305.3	444.0	649.4	949.2	1387.7
Aspartame	294.3	0	0.34	0.67	1.33	2.62	5.16	10.19
Dextrose	198.2	0	191.8	277.5	403.7	590.4	862.9	1261.5
Erythritol	122.1	0	311.2	450.5	655.2	958.2	1400.5	2047.5
Fructose	180.2	0	210.9	305.3	444.0	649.4	949.2	1387.7
Luo han guo	1287.4	0	0.08	0.15	0.30	0.60	1.18	2.33
Maltitol	344.3	0	110.4	159.7	232.4	339.8	496.7	726.1
Mannitol	182.2	0	208.6	301.9	439.1	642.2	938.5	1372.1
Mixture *	–	0	145.5	210.6	306.3	448.0	654.7	957.2
Palatinose	342.2	0	111.0	184.0	309.7	517.1	870.6	1460.7
Sorbitol	182.2	0	208.6	301.9	439.1	642.2	938.5	1372.1
Stevia	967.0	0	0.04	0.09	0.18	0.36	0.75	1.55
Sucralose	397.6	0	0.25	0.51	1.04	2.12	4.33	8.80
Sucrose	342.2	0	111.0	160.7	233.8	341.9	499.7	730.6
Xylitol	152.2	0	249.7	361.4	525.6	768.7	1123.5	1642.6

* Molarity is calculated based on the average molarity of sucrose and allulose in a 1:1 mixture (weight basis)

Table S2. Fitting parameters for dose-response of sweeteners using the Hill equation.

Sweetener	Min. Sweetness R_{min} *	Max. Sweetness R_{max}	$LOGEC_{50}$	Hill Slope
Acesulfame-K	0	24.2	0.050	10.46
Allulose	0	28.3	12.51	0.098
Aspartame	0	29.7	0.066	8.90
Dextrose	0	35.8	15.90	0.057
Erythritol	0	33.1	14.14	0.093
Fructose	0	41.7	-55.78	0.039
Luo han guo	0	30.8	-0.42	4.08
Maltitol	0	35.0	11.57	0.080
Mannitol	0	29.9	15.14	0.13
Mixture ǂ	0	33.0	11.74	0.12
Palatinose	0	32.6	28.95	0.056
Sucralose	0	35.9	-0.34	5.59
Sorbitol	0	33.8	15.43	0.10
Stevia	0	23.7	0.025	23.78
Sucrose	0	37.6	3.60	0.050
Xylitol	0	34.5	10.46	0.11

ǂ 1:1 sucrose–allulose mixture (weight basis); * The parameter 'Rmin' was constrained to zero prior to fitting
